# Supplementary material for: Dopamine System, NMDA Receptor and EGF Family Expressions in Brain Structures of Bl6 and 129Sv Strains Displaying Different Behavioral Adaptation
Source: Brain Sci. 2021 May 29;11(6):725. doi: 10.3390/brainsci11060725 (PMC8227283; doi:10.3390/brainsci11060725)
Supplement: Supplementary file 1 [file brainsci-11-00725-s001.zip › brainsci-1218007-SI.pdf]

Supplementary Material

Dopamine System, NMDA Receptor and EGF Family Expressions in the Brain of Bl6 and 129Sv Strains Related to Different Behavioral Adaptation

Jane Varul [1,2*], Kattri-Liis Eskla[1,2], Maria Piirsalu[1,2], Jürgen Innos [1,2], Mari-Anne Philips [1,2], Tanel Visnapuu [1,2], Mario Plaas [1,2,3] and Eero Vasar [1,2]

[1] Department of Physiology, Institute of Biomedicine and Translational Medicine, University of Tartu, 19 Ravila Street, Tartu, 50411, Estonia.
[2] Center of Excellence for Genomics and Translational Medicine, Institute of Biomedicine and Translational Medicine, University of Tartu, 19 Ravila Street, Tartu, 50411, Estonia.
[3] Laboratory Animal Center, Institute of Biomedicine and Translational Medicine, University of Tartu, 14B Ravila Street, Tartu, 50411, Estonia.

* Correspondence:

Jane Varul

jane.varul@ut.ee

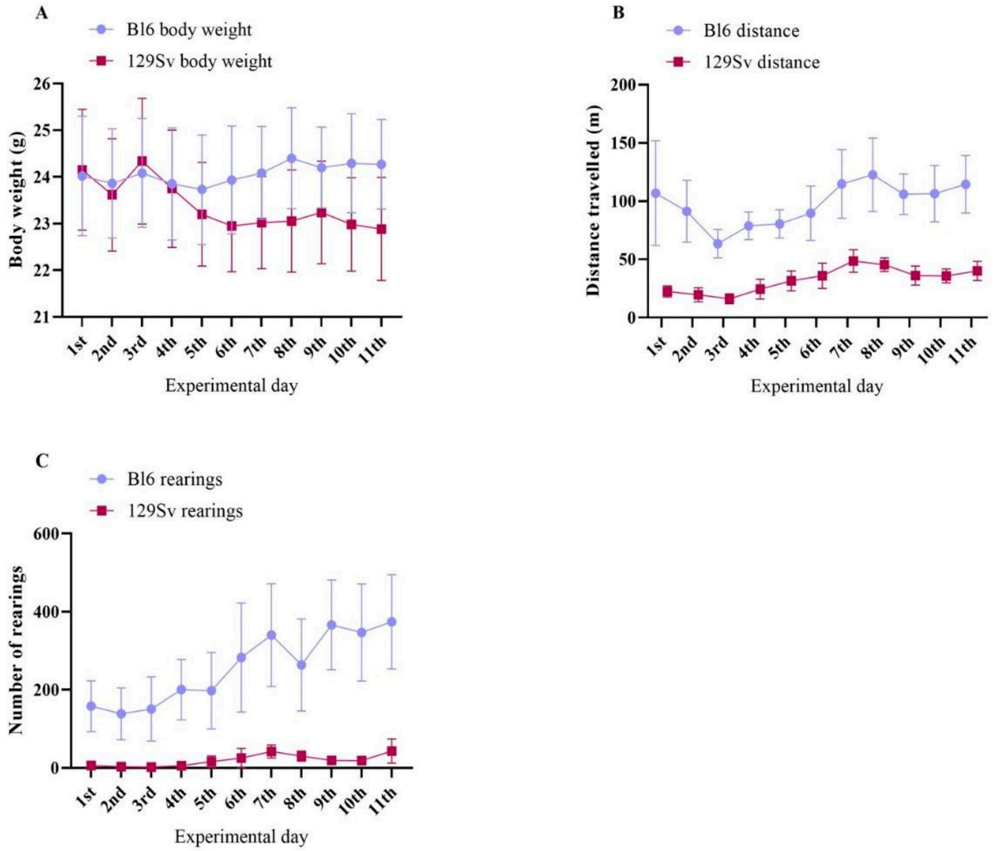

Figure S1. Time course of body weight change and behavioral effects in Bl6 and 129Sv during repeated saline administration (mean and 95% confidence interval). Body weight (**A**), distance travelled (**B**) and number of rearings (**C**) during experimental period. Number of animals in each group varied from 7 to 14.

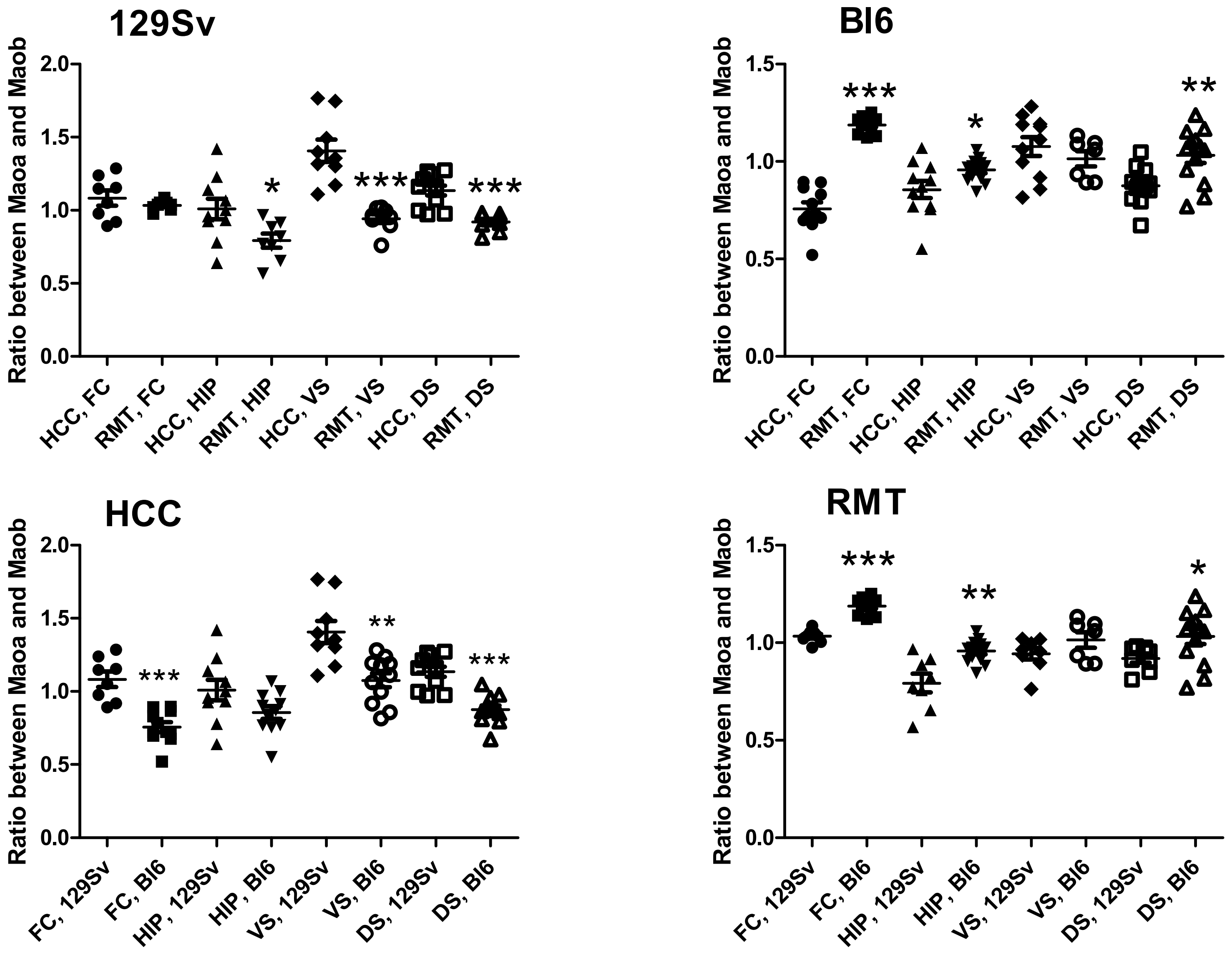

129Sv
Bl6
HCC
RMT
Ratio between Maoa and Maob
2.0
1.5
1.0
0.5
0.0
HCC, FC
RMT, FC
HCC, HIP
RMT, HIP
HCC, VS
RMT, VS
HCC, DS
RMT, DS
FC, 129Sv
FC, Bl6
HIP, 129Sv
HIP, Bl6
VS, 129Sv
VS, Bl6
DS, 129Sv
DS, Bl6
*
**
***

Figure S2. Comparison of ratio between *Maoa* and *Maob* genes in the brain structures of 129Sv and Bl6 mice calculated for HCC and RMT interventions.

HCC - home-cage controls; RMT - repeated motility testing. FC - frontal cortex; HIP - hippocampus; VS - ventral striatum; DS - dorsal striatum Student's t-test (two-tailed): * - $p < 0.05$; ** $p \le 0.01$, *** $p \le 0.001$ compared to the respective HCC group or compared to 129Sv mice. Number of animals in each group varied from 8 to 12.

Table S1

Two-way ANOVA [strain (Bl6 or 129Sv) x environment (HCC or RMT)] summary table of gene expression levels (log_2 values, mean and range) in Bl6 and 129Sv strain of HCC and RMT interventions. Bonferroni *post-hoc* test was used for multiple comparisons. * Statistically significant difference between Bl6 and 129Sv in HCC or RMT batches; # Statistically significant difference between HCC and RMT in Bl6 strain. @ Statistically significant difference between HCC and RMT in 129Sv strain. * $p \leq 0.05$; ** $p \leq 0.01$; *** $p \leq 0.001$; **** $p \leq 0.0001$

Genes	HCC		RMT		Two-way ANOVA		
	Bl6 Mean (min – max)	129Sv Mean (min – max)	Bl6 Mean (min – max)	129Sv Mean (min – max)		*F*	*p*
**Frontal cortex**							
Egf	- 6.85	- 7.20	- 7.04	- 6.34*@@	**Strain**	1.15	0.29
	(- 7.61 – (- 6.05))	(- 7.80 – (- 6.54))	(- 7.66 – (- 5.76))	(- 7.06 – (- 5.35))	**Environment**	4.39	0.043
					**Strain x environment**	10.7	0.002
Tgfα	- 3.77	- 4.70****	- 3.98	- 3.74@@@@	**Strain**	9.24	0.004
	(- 4.11 – (- 3.36))	(- 5.45 – (- 4.31))	(- 4.82 – (- 3.30))	(- 4.49 – (- 3.39))	**Environment**	10.7	0.002
					**Strain x environment**	26.6	δ 0.0001
Hb-Egf	- 5.07	- 5.44	- 4.97	- 4.89@@@	**Strain**	4.07	0.051
	(- 5.32 – (- 4.48))	(- 5.72 – (- 5.17))	(- 5.30 – (- 4.49))	(- 5.10 – (- 4.59))	**Environment**	20.3	δ 0.0001
					**Strain x environment**	9.48	0.004
Nrg1	- 3.13	- 3.44	- 3.87####	- 3.42*	**Strain**	0.40	0.53
	(- 3.51 – (- 2.55))	(- 4.06 – (- 2.83))	(- 4.47 – (- 3.11))	(- 3.69 – (- 3.21))	**Environment**	10.7	0.002
					**Strain x environment**	11.9	0.001

Nrg2	- 5.67<br>(- 6.07 – (- 5.03))	- 5.54<br>(- 5.86 – (- 5.27))	- 5.25#<br>(- 5.79 – (- 4.55))	- 4.45****@@@@<br>(- 4.79 – (- 4.12))	**Strain**<br>**Environment**<br>**Strain x environment**	22.4<br>62.7<br>15.5	δ 0.0001<br>δ 0.0001<br>0.0004
Nrg3	- 3.04<br>(- 3.30 – (- 2.68))	- 3.07<br>(- 3.31 – (- 2.75))	- 3.06<br>(- 3.64 – (- 2.60))	- 2.82<br>(- 3.19 – (- 2.41))	**Strain**<br>**Environment**<br>**Strain x environment**	1.67<br>1.97<br>2.65	0.20<br>0.17<br>0.11
Erbb1	- 3.92<br>(- 4.07 – (- 3.67))	- 3.94<br>(- 4.23 – (- 3.53))	- 4.04<br>(- 4.79 – (- 3.49))	- 3.67*<br>(- 3.94 – (- 3.46))	**Strain**<br>**Environment**<br>**Strain x environment**	4.49<br>0.78<br>5.89	0.041<br>0.38<br>0.020
Erbb2	- 1.97<br>(- 2.30 – (- 1.43))	- 1.66<br>(- 2.25 – (- 1.19))	- 2.19<br>(- 2.98 – (- 0.59))	- 1.51*<br>(- 2.33 – (- 0.90))	**Strain**<br>**Environment**<br>**Strain x environment**	10.2<br>0.049<br>1.47	0.003<br>0.83<br>0.23
Erbb3	- 5.42<br>(- 5.95 – (- 4.76))	- 5.83<br>(- 6.46 – (- 5.34))	- 5.23<br>(- 6.52 – (- 4.34))	- 5.02@@<br>(- 5.66 – (- 4.41))	**Strain**<br>**Environment**<br>**Strain x environment**	0.45<br>11.7<br>4.64	0.51<br>0.002<br>0.038
Erbb4	- 1.97<br>(- 2.30 – (- 1.43))	- 1.66<br>(- 2.25 – (- 1.19))	- 2.18<br>(- 2.78 – (- 1.66))	- 1.52****<br>(- 1.74 – (- 1.36))	**Strain**<br>**Environment**<br>**Strain x environment**	25.3<br>0.13<br>3.57	δ 0.0001<br>0.72<br>0.067
Grin1	0.90<br>(0.61 – 1.37)	0.81<br>(0.058 – 1.27))	0.56#<br>(- 0.065 – 1.00)	0.88*<br>(0.62 – 1.07)	**Strain**<br>**Environment**<br>**Strain x environment**	1.09<br>1.63<br>8.43	0.30<br>0.21<br>0.006
Grin2a	- 0.39<br>(- 0.66 – (- 0.016))	- 0.62<br>(- 0.94 – (- 0.14))	- 0.70<br>(- 1.34 – (- 0.15))	- 0.47<br>(- 0.71 – (- 0.23))	**Strain**<br>**Environment**<br>**Strain x environment**	0.000<br>0.65<br>6.27	1.00<br>0.43<br>0.017
Grin2b	0.24<br>(0.009 – 0.67)	- 0.014<br>(- 0.28 – 0.29)	- 0.05#<br>(- 0.46 – 0.33)	0.19<br>(- 0.12 – 0.48)	**Strain**<br>**Environment**<br>**Strain x environment**	0.004<br>0.36<br>13.1	0.95<br>0.55<br>0.0009
Srr	- 0.52<br>(- 0.80 – (- 0.027))	- 0.65<br>(- 1.01 – 0.36)	- 1.16####<br>(- 1.71 – (- 0.71))	- 0.93<br>(- 1.09 – (- 0.79))	**Strain**<br>**Environment**<br>**Strain x environment**	0.52<br>45.8<br>7.1	0.47<br>δ 0.0001<br>0.01

Drd1	- 4.73	- 4.60	- 4.76	- 4.19	**Strain**	4.26	0.046
	(- 5.21 – (- 4.35))	(- 5.16 – (- 4.09))	(- 5.66 – (- 4.18))	(- 4.80 – (1.87))	**Environment**	1.23	0.27
					**Strain x environment**	1.76	0.19
Drd2	- 5.90	- 5.70	- 5.63	- 5.16	**Strain**	1.46	0.24
	(- 6.72 – (- 4.05))	(- 6.97 – (- 4.67))	(- 6.61 – (- 4.68))	(- 5.47 – (- 4.80))	**Environment**	2.40	0.13
					**Strain x environment**	1.05	0.31
Drd5	- 5.39	- 5.62	- 5.47	- 5.39	**Strain**	0.75	0. 39
	(- 5.72 – (- 4.94))	(- 6.83 – (- 5.10))	(- 6.16 – (- 5.18))	(- 5.78 – (- 5.06))	**Environment**	0.85	0.36
					**Strain x environment**	3.48	0.07
Th	- 4.44	- 4.55	- 3.93	- 1.85	**Strain**	1.54	0.22
	(- 7.18 – (- 0.99))	(- 7.21 – (- 2.32))	(- 6.28 – 0.061)	(- 5.24 – 0.17)	**Environment**	4.61	0.039
					**Strain x environment**	3.45	0.072
Dat	- 6.50	- 7.43	- 6.90	*- 5.02@*	**Strain**	0.70	0.41
	(- 7.84 – (- 3.89))	(- 9.83 – (- 5.67))	(- 9.18 – (- 3.72))	*(- 8.23 – (- 3.37))*	**Environment**	3.11	0.088
					**Strain x environment**	6.00	0.020
Comt	- 3.93	- 4.03	- 3.40####	- 3.12**@@@@	**Strain**	3.41	0.07
	(- 4.22 – (- 3.56))	(- 4.28 – (- 3.69))	(- 4.04 – (- 2.43))	(- 3.36 – (- 3.17))	**Environment**	102.4	δ 0.0001
					**Strain x environment**	11.51	0.0017
Maoa	- 3.87	- 3.84	- 4.16##	- 3.89**	**Strain**	7.36	0.01
	(- 4.06 – (- 3.58))	(- 4.16 – (- 3.58))	(- 4.60 – (- 3.77))	(- 4.13– (- 3.62))	**Environment**	9.54	0.004
					**Strain x environment**	4.76	0.036
Maob	- 3.45	- 4.05****	- 3.51	- 3.77	**Strain**	32.7	δ 0.0001
	(- 3.79 – (- 3.07))	(- 4.80 – (- 3.80))	(- 3.66 – (- 3.01))	(- 3.98 – (- 3.68))	**Environment**	2.24	0.14
					**Strain x environment**	5.16	0.03

Hippocampus							
Egf	- 6.94<br>(-7.29 – (- 6.46))	- 7.25<br>(- 7.64 – (- 6.83))	- 7.50###<br>(- 7.91 – (- 6.70))	- 7.02**<br>(- 7.76 – (- 6.66))	**Strain**<br>**Environment**<br>**Strain x environment**	0.71<br>2.67<br>15.03	0.40<br>0.11<br>0.00042
Tgfα	- 2.74<br>(- 3.02 – (- 2.47))	- 2.71<br>(- 3.06 – (- 2.41))	- 2.78<br>(- 2.97 – (- 2.52))	- 3.01<br>(- 4.25 – (- 2.57))	**Strain**<br>**Environment**<br>**Strain x environment**	1.32<br>3.42<br>2.22	0.26<br>0.072<br>0.15
Hb-Egf	- 4.97<br>(- 5.21 – (- 4.80))	- 4.99<br>(- 5.47 – (- 4.47))	- 5.00<br>(- 5.34 – (- 4.68))	- 5.21<br>(- 5.58 – (- 4.85))	**Strain**<br>**Environment**<br>**Strain x environment**	3.00<br>3.10<br>1.96	0.091<br>0.087<br>0.17
Nrg1	- 4.42<br>(- 4.73 – (- 4.07))	- 3.81**<br>(- 4.50 – (- 3.03))	- 3.05####<br>(- 3.45 – (- 2.50))	- 2.13****@@@@<br>(- 2.89 – (- 1.19))	**Strain**<br>**Environment**<br>**Strain x environment**	39.5<br>159.0<br>1.63	δ 0.0001<br>δ 0.0001<br>0.21
Nrg2	- 5.78<br>(- 6.08 – (- 5.46))	- 5.53<br>(- 5.74 – (- 5.24))	- 5.64<br>(- 6.25 – (- 5.27))	- 5.11***@@@<br>(- 5.42 – (- 4.75))	**Strain**<br>**Environment**<br>**Strain x environment**	18.7<br>16.0<br>5.11	δ 0.0001<br>0.0003<br>0.030
Nrg3	- 3.34<br>(- 3.57 – (- 3.11))	- 3.17<br>(- 3.96 – (- 2.20))	- 2.04####<br>(- 2.32 – (- 1.60))	- 1.69@@@@<br>(- 1.91 – (- 1.01))	**Strain**<br>**Environment**<br>**Strain x environment**	7.08<br>208<br>0.99	0.011<br>δ 0.0001<br>0.33
Erbb1	- 4.15<br>(- 4.42 – (- 3.74))	- 3.97<br>(- 5.04 – (- 3.42))	- 4.36<br>(- 4.66 – (- 4.11))	- 4.05**<br>(- 4.24 – (- 3.80))	**Strain**<br>**Environment**<br>**Strain x environment**	13.89<br>4.73<br>1.10	0.0007<br>0.036<br>0.30

Erbb2	- 6.37<br>(- 6.85 – (- 6.05))	- 5.97<br>(- 6.95 – (- 5.08))	- 6.24<br>(- 6.75 – (- 5.85))	- 5.83<br>(- 6.54 – (- 5.21))	**Strain**<br>**Environment**<br>**Strain x environment**	12.9<br>0.83<br>0.030	0.001<br>0.37<br>0.87
Erbb3	- 4.83<br>(- 5.24 – (- 4.62))	- 4.88<br>(- 5.31 – (- 4.64))	- 4.95<br>(- 5.28 – (- 4.61))	- 5.11<br>(- 5.86 – (- 4.66))	**Strain**<br>**Environment**<br>**Strain x environment**	1.43<br>3.73<br>0.33	0.24<br>0.061<br>0.57
Erbb4	- 2.34<br>(- 2.69 – (- 1.97))	- 1.72****<br>(- 1.91 – (- 1.44))	- 2.27<br>(- 2.53 – (- 2.05))	- 1.54****<br>(- 1.75 – (- 1.17))	**Strain**<br>**Environment**<br>**Strain x environment**	57.7<br>5.22<br>1.76	δ 0.0001<br>0.028<br>0.19
Grin1	0.63<br>(0.29 – 0.98)	1.00*<br>(0.71 – 1.44)	0.50<br>(0.29 – 0.68)	0.81*<br>(0.41 – 1.05)	**Strain**<br>**Environment**<br>**Strain x environment**	16.6<br>2.40<br>0.061	0.0002<br>0.13<br>0.81
Grin2a	- 0.12<br>(- 0.41 – (- 0.071))	0.05<br>(- 0.70 – 1.17)	0.38###<br>(0.16 – 0.63)	0.52@@@<br>(- 0.42 – 1.10)	**Strain**<br>**Environment**<br>**Strain x environment**	1.64<br>40.7<br>0.13	0.21<br>δ 0.0001<br>0.762
Grin2b	0.32<br>(0.045 – 0.60)	0.63**<br>(0.23 – 1.21)	0.16<br>(- 0.14 – 0.53)	0.68****<br>(0.38 – 1.30)	**Strain**<br>**Environment**<br>**Strain x environment**	40.3<br>0.85<br>2.53	δ 0.0001<br>0.36<br>0.12
Srr	- 0.96<br>(- 1.18 – (- 0.78))	- 0.73<br>(- 0.84 – (-0.65))	- 0.70##<br>(- 0.90 – (- 0.41))	- 0.48@@<br>(- 0.88 – (- 0.15))	**Strain**<br>**Environment**<br>**Strain x environment**	7.44<br>13.0<br>0.048	0.010<br>0.0009<br>0.83
Drd1	- 4.86<br>(- 5.16 – (- 4.52))	- 4.25**<br>(- 4.93 – (- 3.79))	- 4.72<br>(- 5.31 – (- 4.38))	- 3.74****<br>(- 4.34 – (- 2.34))	**Strain**<br>**Environment**<br>**Strain x environment**	38.7<br>6.55<br>2.18	δ 0.0001<br>0.015<br>0.15
Drd2	- 4.65<br>(- 5.18 – (- 3.84))	- 4.11*<br>(- 5.34 – (- 3.58))	- 4.66<br>(- 5.26 – (- 3.95))	- 4.33<br>(- 4.89 – (- 1.80))	**Strain**<br>**Environment**<br>**Strain x environment**	10.5<br>0.77<br>0.68	0.003<br>0.39<br>0.41
Drd3	- 8.56<br>(- 9.01 – (- 7.97))	- 8.93<br>(- 9.45 – (- 8.18))	- 7.44###<br>(- 8.09 – (- 6.71))	- 7.35@@@@<br>(- 8.54 – (- 5.75))	**Strain**<br>**Environment**<br>**Strain x environment**	0.51<br>47.7<br>1.38	0.48<br>δ 0.0001<br>0.25

Drd5	- 3.29<br>(- 3.60 – (- 2.91))	- 3.05<br>(- 3.44 – (- 2.31))	- 2.72###<br>(- 3.21 – (- 2.50))	- 2.41@@@<br>(- 3.24 – (- 1.74))	**Strain**	9.44	0.0041
					**Environment**	44.7	<0.0001
					**Strain x environment**	0.18	0.68
Th	- 7.87<br>(- 8.28 – (- 7.37))	- 7.88<br>(- 8.44 – (- 7.41))	- 7.73<br>(- 8.31 – (- 6.58))	- 7.71<br>(- 8.72 – (- 6.47))	**Strain**	0.002	0.96
					**Environment**	1.07	0.31
					**Strain x environment**	0.013	0.91
DAT	-8.55<br>(-9.02 – (-7.97))	-8.74<br>(-9.42 – (-7.92))	-8.25<br>(-9.67 – (-7.14))	-8.52<br>(-10.46 – (-6.67))	**Strain**	0.89	0.35
					**Environment**	1.18	0.28
					**Strain x environment**	0.01	0.91
Comt	- 3.61<br>(- 3.88 – (- 3.43))	- 3.56<br>(- 3.89 – (- 3.20))	- 3.84#<br>(- 4.04 – (- 3.63))	- 3.67<br>(- 4.05 – (- 3.38))	**Strain**	5.38	0.026
					**Environment**	8.55	0.006
					**Strain x environment**	0.50	0.48
Maoa	- 3.54<br>(- 3.71 – (- 3.28))	- 3.74<br>(- 4.52 – (- 3.41))	- 2.77####<br>(- 3.07 – (- 2.58))	- 2.64@@@@<br>(- 2.91 – (- 2.67)	**Strain**	0.27	0.61
					**Environment**	168	δ 0.0001
					**Strain x environment**	5.15	0.029
Maob	- 3.30<br>(- 3.57 – (- 2.86))	- 3.72**<br>(- 4.50 – (- 3.15))	- 2.90##<br>(- 3.24 – (- 2.67))	- 3.37**<br>(- 4.02 – (- 3.01))	**Strain**	28.0	δ 0.0001
					**Environment**	19.7	δ 0.0001
					**Strain x environment**	0.078	0.78
**Ventral striatum**							
Egf	- 6.80<br>(- 7.26 – (- 6.16))	- 7.27*<br>(- 7.54 – (- 7.03))	- 6.50<br>(- 7.05 – (- 5.98))	- 6.24@@@@<br>(- 6.95 – (- 5.73))	**Strain**	0.78	0.39
					**Environment**	26.8	δ 0.0001
					**Strain x environment**	7.86	0.009

Tgfα	- 1.96<br>(- 2.38 – (- 1.43))	- 2.35<br>(- 3.14 – (- 1.88))	- 1.80<br>(- 2.22 – (- 1.20))	- 2.27<br>(- 3.06 – (- 1.71))	**Strain**<br>**Environment**<br>**Strain x environment**	10.2<br>0.72<br>0.081	0.003<br>0.40<br>0.78
Hb-Egf	- 4.34<br>(- 4.70 – (- 3.87))	- 4.76**<br>(- 5.13 – (- 4.45))	- 4.34<br>(- 4.71 – (- 3.89))	- 4.55<br>(- 5.09 – (- 4.16))	**Strain**<br>**Environment**<br>**Strain x environment**	13.9<br>1.58<br>1.53	0.0008<br>0.22<br>0.23
Nrg1	- 2.64<br>(- 3.05 – (- 2.11))	- 2.89<br>(- 3.14 – (- 2.56))	- 3.28###<br>(- 3.46 – (- 2.89))	- 3.40@@<br>(- 3.89 – (- 2.86))	**Strain**<br>**Environment**<br>**Strain x environment**	4.13<br>38.9<br>0.46	0.051<br>δ 0.0001<br>0.50
Nrg2	- 4.43<br>(- 4.82 – (- 4.03))	- 4.49<br>(- 4.84 – (- 4.10)	- 4.62<br>(- 4.94 – (- 4.44))	- 4.67<br>(- 5.14 – (- 4.23))	**Strain**<br>**Environment**<br>**Strain x environment**	0.44<br>4.36<br>0.002	0.51<br>0.045<br>0.96
Nrg3	- 3.84<br>(- 4.16 – (- 3.14))	- 3.97<br>(- 4.33 – (- 3.66))	- 3.18###<br>(- 3.69 – (- 2.84))	- 3.01@@@@<br>(- 3.25 – (- 2.34))	**Strain**<br>**Environment**<br>**Strain x environment**	0.030<br>68.7<br>2.35	0.86<br>δ 0.0001<br>0.14
Erbb1	- 3.63<br>(- 4.11 – (- 3.20))	- 3.84<br>(- 4.30 – (- 3.61))	- 3.34<br>(- 3.69 – (- 2.94))	- 3.38@@<br>(- 3.71 – (- 3.04))	**Strain**<br>**Environment**<br>**Strain x environment**	2.03<br>19.1<br>1.05	0.17<br>δ 0.0001<br>0.31
Erbb2	- 5.92<br>(- 6.24 – (- 5.43))	- 6.07<br>(- 6.46 – (- 5.73))	- 5.68<br>(- 6.70 – (- 5.27))	- 5.78<br>(- 6.42 – (- 5.40))	**Strain**<br>**Environment**<br>**Strain x environment**	1.19<br>5.33<br>0.034	0.28<br>0.028<br>0.86
Erbb3	- 4.19<br>(- 4.76 – (- 3.70))	- 4.55<br>(- 4.77 – (- 4.40))	- 4.52<br>(- 5.18 – (- 4.30))	- 4.83<br>(- 5.46 – (- 4.35))	**Strain**<br>**Environment**<br>**Strain x environment**	9.75<br>8.20<br>0.058	0.004<br>0.007<br>0.81
Erbb4	- 1.18<br>(- 1.51 – (- 0.41))	- 1.12<br>(- 1.38 – (- 0.91))	- 1.31<br>(- 2.29 – (- 0.95))	- 1.15<br>(- 1.48 – (- 0.87))	**Strain**<br>**Environment**<br>**Strain x environment**	0.20<br>1.82<br>1.16	0.66<br>1.19<br>0.29

Grin1	0.70<br>(0.36 – 1.34)	0.79<br>(- 0.088 – 0.94)	0.30#<br>(- 0.099 – 0.70)	0.39@<br>(- 0.029 – 0.72)	**Strain**	0.000	1.00
					**Environment**	10.5	0.003
					**Strain x environment**	0.66	0.42
Grin2a	- 1.07<br>(- 1.50 – (- 0.41))	- 1.29<br>(- 2.05 – (- 0.89))	- 1.20<br>(- 1.41 – (- 0.82))	- 1.20<br>(- 1.92 – (- 0.77))	**Strain**	0.83	0.37
					**Environment**	0.026	0.87
					**Strain x environment**	0.71	0.041
Grin2b	0.12<br>(- 0.30 – 0.70)	0.064<br>(- 0.39 – 0.46)	- 0.078<br>(- 0.32 – 0.17)	- 0.18<br>(- 0.73 – 0.24)	**Strain**	0.63	0.43
					**Environment**	4.75	0.037
					**Strain x environment**	0.048	0.83
Srr	- 0.93<br>(- 1.21 – (- 0.41))	- 0.98<br>(- 1.42 – (- 0.68))	- 0.85<br>(- 1.06 – (- 0.66))	- 0.93<br>(- 1.35 – (- 0.49))	**Strain**	0.58	0.45
					**Environment**	0.55	0.46
					**Strain x environment**	0.015	0.90
Drd1	0.23<br>(- 0.12 – 0.67)	0.18<br>(- 0.63 – 0.62)	0.63<br>(0.12 – 1.04)	0.41<br>(- 0.83 – 1.11)	**Strain**	0.76	0.39
					**Environment**	3.84	0.059
					**Strain x environment**	0.30	0.59
Drd2	0.97<br>(0.48 – 1.80)	0.85<br>(0.036 – 1.23)	0.16##<br>(- 0.39 – 1.20)	- 0.18@@@<br>(- 1.18 – 0.52)	**Strain**	1.92	0.18
					**Environment**	31.3	δ 0.0001
					**Strain x environment**	0.46	0.50
Drd3	- 3.06<br>(- 3.45 – (- 2.34))	- 3.28<br>(- 3.98 – (- 2.84))	- 3.28<br>(- 6.29 – (- 2.87))	- 3.37<br>(- 4.55 – (- 2.56))	**Strain**	0.063	0.80
					**Environment**	2.57	0.12
					**Strain x environment**	1.49	0.23
Drd4	- 8.19<br>(- 8.89 – (- 7.26))	- 8.72<br>(- 9.57 – (- 7.85))	- 9.66####<br>(- 10.1 – (- 8.93))	- 9.94@@@<br>(- 10.5 – (- 9.35))	**Strain**	4.85	0.036
					**Environment**	54.2	δ 0.0001
					**Strain x environment**	0.46	0.50
Drd5	- 4.73<br>(- 5.17 – (- 4.21))	- 4.60<br>(- 5.06 – (- 4.22))	- 4.66<br>(- 5.09 – (- 4.26))	- 4.47<br>(- 4.78 – (- 4.19))	**Strain**	2.68	0.11
					**Environment**	0.92	0.34
					**Strain x environment**	0.095	0.76
Th	- 4.71<br>(- 5.04 – (- 3.93))	- 4.86<br>(- 5.35 – (- 4.29))	- 4.12#<br>(- 4.84 – (- 3.69))	- 4.13@@@<br>(- 4.64 – (- 3.66))	**Strain**	0.94	0.34
					**Environment**	26.5	δ 0.0001
					**Strain x environment**	0.86	0.36

Comt	- 3.56<br>(- 3.74 – (- 3.11))	- 3.61<br>(- 3.97 – (- 3.46))	- 0.85####<br>(- 1.06 – (- 0.66))	- 0.93@@@@<br>(- 1.35 – (- 0.49))	**Strain**<br>**Environment**<br>**Strain x environment**	0.77<br>1373<br>0.039	0.39<br>δ 0.0001<br>0.84
Dat	- 6.91<br>(- 7.62 – (- 6.31))	- 7.16<br>(- 7.57 – (- 6.13))	- 7.98##<br>(- 9.48 – (- 7.07))	- 7.79<br>(- 8.09 – -(7.31))	**Strain**<br>**Environment**<br>**Strain x environment**	0.015<br>16.8<br>1.14	0.90<br>0.0003<br>0.30
Maoa	- 3.29<br>(- 3.42 – (- 3.08))	- 3.24<br>(- 3.48 – (- 3.06))	- 2.59####<br>(- 2.92 – (2.40))	- 2.56@@@@<br>(- 2.80 – (- 2.32))	**Strain**<br>**Environment**<br>**Strain x environment**	0.69<br>197<br>0.006	0.41<br>δ 0.0001<br>0.94
Maob	- 3.41<br>(- 3.65 – (- 3.09))	- 3.72**<br>(- 4.02 – (- 3.50))	- 2.57####<br>(- 2.79 – (2.31))	- 2.72@@@@<br>(- 3.05 – (- 2.42))	**Strain**<br>**Environment**<br>**Strain x environment**	15.2<br>207<br>2.31	0.0005<br>δ 0.0001<br>0.14
**Dorsal striatum**							
Egf	- 6.61<br>(- 6.98 – (- 5.76))	- 7.18*<br>(- 7.81 – (- 6.47))	- 6.58<br>(- 7.24 – (- 6.05))	- 5.98**@@@@<br>(- 7.01 – (- 5.38))	**Strain**<br>**Environment**<br>**Strain x environment**	0.010<br>23.9<br>21.7	0.92<br>δ 0.0001<br>δ 0.0001
Tgfα	- 0.71<br>(- 1.22 – (- 0.33))	- 0.96<br>(- 1.33 – (- 0.67))	- 0.12##<br>(- 1.40 – 0.66)	- 0.37@<br>(- 1.50 – 0.40)	**Strain**<br>**Environment**<br>**Strain x environment**	3.53<br>20.1<br>0.000	0.068<br>δ 0.0001<br>0.99
Hb-Egf	- 4.18<br>(- 4.44 – (- 3.73))	- 4.43<br>(- 4.91 – (- 4.18))	- 2.73###<br>(- 4.78 – (- 2.27))	- 3.10@@@<br>(- 8.17 – (- 2.75))	**Strain**<br>**Environment**<br>**Strain x environment**	12.7<br>247<br>0.47	0.001<br>δ 0.0001<br>0.50

Nrg1	- 2.58 (- 3.01 – (- 1.80))	- 2.89* (- 3.08 – (- 2.63))	- 2.22 (- 2.96 – (- 1.84))	- 2.70** (- 3.41 – (- 2.31))	**Strain**	20.1	δ 0.0001
					**Environment**	6.32	0.016
					**Strain x environment**	0.30	0.59
Nrg2	- 3.98 (- 4.35 – (- 3.44))	- 3.97 (- 4.26 – (- 3.75))	- 3.35## (- 4.15 – (- 2.82))	- 3.39@ (- 4.56 – (- 2.34))	**Strain**	0.013	0.091
					**Environment**	26.2	δ 0.0001
					**Strain x environment**	0.036	0.85
Nrg3	- 3.88 (- 4.27 – (- 3.26))	- 4.30 (- 4.75 – (- 3.42))	- 2.35#### (- 3.19 – (- 1.66))	- 2.52@@@@ (- 3.27 – (- 1.78))	**Strain**	5.19	0.028
					**Environment**	160	δ 0.0001
					**Strain x environment**	0.95	0.34
Erbb1	- 3.80 (- 4.07 – (- 3.18))	- 3.80 (- 4.12 – (- 3.56))	- 3.18#### (- 3.51 – (- 2.77))	- 3.17@@@@ (- 3.87 – (- 2.82))	**Strain**	0.005	0.95
					**Environment**	63.6	δ 0.0001
					**Strain x environment**	0.003	0.95
Erbb2	- 5.44 (- 5.93 – (- 4.51))	- 5.22 (- 5.95 – (- 4.26))	- 4.83## (- 5.30 – (- 4.40))	- 4.91 (- 5.49 – (- 4.35))	**Strain**	0.339	0.56
					**Environment**	13.8	0.0006
					**Strain x environment**	1.42	0.24
Erbb3	- 4.06 (- 4.39 – (- 3.59))	- 4.56 (- 4.78 – (- 4.29))	- 3.33### (- 4.35 – (- 2.27))	- 3.82@@ (- 4.73 – (- 3.00))	**Strain**	13.2	0.0008
					**Environment**	29.2	δ 0.0001
					**Strain x environment**	0.000	0.99
Erbb4	- 2.08 (- 2.41 – (- 0.62))	- 1.95 (- 2.20 – (- 1.63))	- 2.22 (- 4.35 – (- 1.66))	- 1.75 (- 2.12 – (- 1.27))	**Strain**	2.31	0.14
					**Environment**	0.054	0.82
					**Strain x environment**	2.46	0.13
Grin1	1.13 (0.56 – 1.40)	1.13 (0.90 – 1.39)	2.82#### (1.88 – 3.50)	2.84@@@@ (2.84 – 3.68)	**Strain**	0.004	0.95
					**Environment**	130	δ 0.0001
					**Strain x environment**	0.002	0.96
Grin2a	- 0.68 (- 1.20 – (- 0.31))	- 0.83 (- 1.02 – (- 0.55))	0.20#### (- 0.64 – 1.04))	0.17@@@@ (- 0.26 – 0.60)	**Strain**	0.57	0.46
					**Environment**	57.5	δ 0.0001
					**Strain x environment**	0.24	0.63
Grin2b	0.37 (0.10 – 1.16)	0.33 (0.075 -0.65)	0.65# (- 0.27 – 0.89)	0.74@@@ (- 0.073 – 0.96)	**Strain**	0.09	0.76
					**Environment**	29.3	δ 0.0001
					**Strain x environment**	0.97	0.33

Srr	- 0.35 (- 0.58 – (- 0.094))	- 0.45 (- 0.60 – (- 0.36))	0.017## (- 0.57 – 0.51)	0.096@@@ (- 0.46 – 0.50)	**Strain**	0.018	0.89
					**Environment**	35.8	δ 0.0001
					**Strain x environment**	1.41	0.24
Drd1	1.27 (0.96 – 1.64)	1.30 (0.83 – 1.59)	2.14 #### (1.22 – 2.62)	2.15@@@ (1.82 – 3.13)	**Strain**	0.031	0.86
					**Environment**	112	δ 0.0001
					**Strain x environment**	0.006	0.94
Drd2	1.64 (1.00 – 2.14)	1.82 (1.42 – 2.42)	2.27#### (1.97 – 3.06)	2.44@@@ (1.87 – 3.10)	**Strain**	1.33	0.26
					**Environment**	51.6	δ 0.0001
					**Strain x environment**	0.17	0.68
Drd3	-5.26 (- 6.35 – (- 4.36))	-5.48 (- 6.30 – (- 4.14))	-4.73 (- 5.81 – (- 3.49))	-5.08 (- 6.00 – (- 4.20))	**Strain**	2.39	0.13
					**Environment**	6.44	0.015
					**Strain x environment**	0.12	0.73
Drd5	- 5.02 (- 5.59 – (- 4.71))	- 5.27 (- 5.56 – (- 4.58))	- 4.64 (- 5.48 – (- 4.18))	- 5.06 (- 6.11 – (- 4.39))	**Strain**	8.92	0.005
					**Environment**	7.09	0.011
					**Strain x environment**	0.61	0.44
Th	- 4.75 (- 5.33 – (- 4.19))	- 4.84 (- 5.24 – (- 4.37))	- 4.27## (- 4.71 – (-3.43))	- 4.53 (- 5.18 – (- 4.03))	**Strain**	2.89	0.097
					**Environment**	14.7	0.0004
					**Strain x environment**	0.66	0.42
Comt	- 3.50 (- 3.78 – (- 2.97))	- 3.62 (- 3.91 – (- 3.30))	- 4.16### (- 5.10 – (- 3.69))	- 4.12@ (- 5.71 – (- 3.60))	**Strain**	0.11	0.74
					**Environment**	23.7	δ 0.0001
					**Strain x environment**	0.40	0.53
Dat	- 7.45 (- 8.40 – (- 6.74))	- 8.29** (- 9.04 – (- 7.53))	- 7.90 (- 8.92 – (- 6.37))	- 8.28 (- 8.85 – (- 7.82))	**Strain**	11.8	0.002
					**Environment**	1.52	0.23
					**Strain x environment**	1.70	0.20
Maoa	- 3.10 (- 3.33 – (- 2.59))	- 3.16 (- 3.39 – (- 2.92))	- 3.05 (- 3.44 – (- 2.28))	- 3.20 (- 3.69 – (- 2.97))	**Strain**	2.49	0.12
					**Environment**	0.001	0.98
					**Strain x environment**	0.42	0.52

Maob	- 2.90<br>(- 3.10 – (- 2.52))	- 3.34****<br>(- 3.47 – (- 3.14))	- 2.98<br>(- 3.63 – (- 2.71))	- 3.49****<br>(- 3.76 – (- 3.07))	**Strain**	53.1	δ 0.0001
					**Environment**	3.31	0.076
					**Strain x environment**	0.30	0.59
**Midbrain**							
Drd1	- 5.92<br>(- 6.37 – (- 5.48))	- 5.67<br>(- 6.59 – (- 5.05))	- 5.97<br>(- 6.44 – (- 5.48))	- 6.31@@<br>(- 7.24 – (- 5.91))	**Strain**	0.098	0.76
					**Environment**	6.13	0.019
					**Strain x environment**	4.33	0.046
Drd2	- 0.93<br>(- 1.87 – (- 0.31))	- 0.99<br>(- 2.08 – 0.20)	- 1.32<br>(- 2.17 – (- 0.36))	- 0.70<br>(- 1.35 – (- 0.18))	**Strain**	1.96	0.17
					**Environment**	0.069	0.79
					**Strain x environment**	2.88	0.10
Drd3	- 6.93<br>(- 7.45 – (- 6.43))	- 6.69<br>(- 7.30 – (- 5.62))	- 7.11<br>(- 7.77 – (- 6.23))	- 6.51*<br>(- 7.22 – (- 6.02))	**Strain**	6.86	0.014
					**Environment**	0.000	1.00
					**Strain x environment**	1.30	0.26
Drd5	- 5.15<br>(- 5.50 – (- 4.47))	- 5.24<br>(- 5.80 – (- 4.42))	- 5.54<br>(- 6.00 – (- 4.80))	- 5.43<br>(- 5.83 – (- 5.02))	**Strain**	0.009	0.93
					**Environment**	4.94	0.034
					**Strain x environment**	0.56	0.46
Th	- 0.66<br>(- 1.56 – (- 0.27))	- 0.63<br>(- 1.46 – 0.31)	- 1.15<br>(- 2.72 – (- 0.12))	- 0.77<br>(- 1.14 – (- 0.53))	**Strain**	1.17	0.29
					**Environment**	2.81	0.10
					**Strain x environment**	0.84	0.37
Dat	0.77<br>(0.017 – 1.22)	0.93<br>(- 0.022 – 2.05)	0.13<br>(- 1.15 – 1.32)	0.54<br>(0.013 – 0.92)	**Strain**	2.03	0.16
					**Environment**	6.55	0.016
					**Strain x environment**	0.42	0.52
Comt	- 3.67<br>(- 3.97 – (- 3.40))	- 3.87<br>(- 4.36 – (- 3.55))	- 4.29###<br>(- 4.83 – (- 3.99))	- 4.30@@<br>(- 4.73 – (- 3.97))	**Strain**	1.21	0.28
					**Environment**	30.4	δ 0.0001
					**Strain x environment**	0.87	0.36

Table S2. Primers used for qPCR analysis.

Gene (Mouse)	Full gene name	Forward primer (5'3')	Reverse primer (5'3')
**Egf family**			
Egf	epidermal growth factor	TTTGCACAGACAGCCCTG AAG	CAATCCAGTCCAGGGCAAG AC
Tgfα	transforming growth factor alpha	CCAGCATGTGTCTGCCAC TC	ACAATGGAGACCACCACCA G
Hb-Egf	heparin binding EGF like growth factor	TGCAAATGCCTCCCTGGT TAC	CTACAGCCACCACAGCCAA G
Nrg1	neuregulin 1	GGTGGTCGGCATCATGTG TG	GCCATTCGCTATGTTCACC ATG
Nrg2	neuregulin 2	TTCTCACCACTGCTCCACA G	CATGATGCCTGACTGGGAA TCC
Nrg3	neuregulin 3	AGCTTGATGAAGAGCCAT GTCC	AACGACTGGGGAGCTGAA AAAC
Erbb1	epidermal growth factor receptor	ATGCTGTACAACCCCACC AC	TGAGCCATGATCTGTCACC AC
Erbb2	erb–b2 receptor tyrosine kinase 2	CTGCCTGACATCCACAGT GC	AACACACCAGTTGAGCAGG TC

Erbb3	erb–b2 receptor tyrosine kinase 3	TTTCCAACCTGACGACCATCG	ACACGCCCAGCACTAATTTCC
Erbb4	erb–b2 receptor tyrosine kinase 4	TGTGTACGAGCCTGCCCTAG	CAGTGATCCCGTGCCGATTC
**NMDA receptors**			
Grin1	glutamate ionotropic receptor NMDA type subunit 1	GTTCTTCCGCTCCGGCTTTG	CGAACCCATGTCTTATCCAGGTC
Grin2a	glutamate ionotropic receptor NMDA type subunit 2A	TGCAAGTTACACAGCCAACCTG	ATCGGAAAGGCGGAGAATAGTC
Grin2b	glutamate ionotropic receptor NMDA type subunit 2B	TCATTTCTGCTCAGACTCTCACC	TCAATGGATGGGCCAAACTGG
Srr	serine racemase	AGAAGCCCAAAGCCGTAGTTAC	TGCAGTTGGGAGCTGTTTGG
**Dopamine system**			
Drd1	dopamine receptor D1	GAGCAGGACATACGCCATTT	CCTCTCCAAAGCTGAGATGC
Drd2	dopamine receptor D2	TCATTGCCAACCCTGCCTTC	TTGGTGTTGACCCGCTTCC
Drd3	dopamine receptor D3	ACGTGTCCCCAGAGCTTTAC	TGAGGAAGGCTTTGCGGAAC
Drd4	dopamine receptor D4	GACGCCTTTCTTCGTGGTG	TGGTGTAGATGATGGGGTTGAG
Drd5	dopamine receptor D5	GGCTATTTCCAGACCCTTCC	TTGAGTTGGACCGGGATAAA
Th	tyrosine hydroxylase	ACCGCACATTTGCCCAGTTC	ACACAGCCCAAACTCCACAG

Comt	catechol-O-methyltransferase	GAGATGGACCGGAACTTTGA	AGTTGCCAGCACTGAGGTTT
Maoa	monoamine oxidase A	AGCCTACTTCCCTCCTGGTATC	AGCTTCAACTGCACCTTCCATG
Maob	monoamine oxidase B	GAATCTTTGGATGTCCCTGCAC	TGTTGCTGACAAGATGGTGGTC
Dat	dopamine transporter	TGCTGCTCACTCTGGGTATC	GTAGCCAGGACAATGCCAAG
**Housekeeping gene**			
Hprt	hypoxanthine guanine phosphoribosyl transferase	AGTTCTTTGCTGACCTGCTG	ATGTCCCCCGTTGACTGATC

Table S3. Antibody list used for Western blot.

Protein	Primary Ab	Secondary Ab	Host	Catalog No
EGF	1:100	1:1000	Mouse	sc-374255
ERBB1	1:500	1:1000	Mouse	sc-373746
NRG1	1:1000	1:5000	Mouse	sc-393006
NRG2	1:1000	1:5000	Mouse	sc-398594
GRIN1	1:15 000	1:20 000	Rabbit	ab17345
B-actin	1:1000	1:10 000	Mouse	sc-47778
